# Performance Evaluation of *SHOX2* and *RASSF1A* Methylation for the Aid in Diagnosis of Lung Cancer Based on the Analysis of FFPE Specimen

**DOI:** 10.3389/fonc.2020.565780

**Published:** 2020-12-14

**Authors:** Juanhong Shi, Xue Chen, Long Zhang, Xia Fang, Yuting Liu, Xuyou Zhu, Haoyang Zhang, Lichao Fan, Jun Gu, Suxia Zhang, Bin She, Hongxiu Han, Xianghua Yi

**Affiliations:** ^1^ Department of Pathology, Tongji Hospital, Tongji University, Shianghai, China; ^2^ Department of Pulmonary and Critical Care Medicine, Dongfang Hospital Affiliated to Tongji University, Shanghai, China; ^3^ Department of Pulmonary and Critical Care Medicine, Shanghai Pulmonary Hospital Affiliated to Tongji University, Shanghai, China; ^4^ Academic Development, Tellgen Corporation, Shanghai, China

**Keywords:** lung cancer, *SHOX2*, *RASSF1A*, DNA methylation, epigenetic field defect

## Abstract

Emerging molecular diagnostic methods are more sensitive and objective, which can overcome the intrinsic failings of morphological diagnosis. Here, a RT-PCR-based *in vitro* diagnostic test kit (LungMe^®^) was developed and characterized to simultaneously quantify the DNA methylation of *SHOX2* and *RASSF1A* in FFPE tissue specimens. The clinical manifestations were evaluated in 251 FFPE samples with specificity and sensitivity of 90.4 and 89.8%, respectively. Furthermore, the quantitative analysis shows that the degree of *SHOX2* methylation was correlated with the stages of lung cancer, but not in the case of *RASSF1A*. Our observation indicated that the DNA methylation of *SHOX2* and *RASSF1A* may play different roles in cancer development. Comparison of the methylation levels of *SHOX2* and *RASSF1A* between cancer and cancer-adjacent specimens (n = 30), showed they have “epigenetic field defect”. As additional clinical validation, the hypermethylation of *SHOX2* and *RASSF1A* was detected not only in surgical operative specimens, but also in histopathological negative puncture biopsies. *SHOX2* and *RASSF1A* methylation detection can be used to increase sensitivity and NPV, which provide us with a more accurate method of differential diagnosis and are likely to be rapidly applied in clinical examinations.

## Introduction

Lung cancer is the leading cause of cancer-related deaths worldwide (1). Bronchoscopic techniques, sputum cytology, transthoracic needle biopsies, and surgical biopsy have been widely used in the diagnosis of bronchogenic carcinoma (2). The conventional morphological diagnosis including cytological and histological examination could be severely affected by the quality of specimen and the diagnostic level of the individual pathologist (3, 4). Emerging molecular diagnosis methods are more sensitive and objective, which can overcome the intrinsic failings of morphological diagnosis. Among the most promising biomarkers, DNA methylation alterations have emerged as a helpful adjunct in both the diagnosis and staging of lung cancer (5–7).

DNA methylation has played a crucial role in the regulation of gene expression, epigenetic changes, and maintenance of cellular identity, occurring frequently in tumorigenesis (8). In particular, the promoter CpG inland hypermethylation may lead to the transcriptional silencing of tumor suppressor genes, and affect the development of carcinogenesis (9). Previous studies have demonstrated a wide range of DNA methylation abnormalities in lung cancer (5–7).

Relevant pieces of evidence show that the promoter hypermethylation of the short stature homeobox gene two (*SHOX2*) has been identified as diagnostic biomarkers for lung cancer (5, 6, 10). However, the results also indicate that *SHOX2* gene methylation exhibits lower sensitivity for stage I tumors, and the sensitivity to small cell lung cancer (SCLC) and squamous cell carcinoma (SCC) is higher than that of adenocarcinoma (Adenoca). Currently, the RAS association domain family 1, isoform A (*RASSF1A*), has been intensively studied as an additional DNA methylation biomarker in lung cancer. To facilitate the use in a diagnostic setting, an *in vitro* diagnostic test kit LungMe^®^ Assay has been developed and validated for NMPA-marking (China National Medical Products Administration). Previous studies using this assay showed that the combination of *SHOX2* and *RASSF1A* methylation in bronchoalveolar lavage fluid (BALF) produced 71.5–81.0% increase in diagnostic sensitivity and 90–97.4% in specificity, especially in an early stage (11, 12).

As the final step in the diagnosis of lung cancer, histopathological specimens obtained from transbronchial (TBLB), endobronchial biopsy (EBLB), and transthoracic needle aspiration (TTNA) have been identified as good tool for diagnosing and staging of lung cancer, with a sensitivity of 71–98% (2, 13, 14). However, the diagnostic performance depends on individual experience of investigators including bronchoscopists and pathologists. Besides, due to possible sampling error, no test can rule out the risk of missing malignant lesions. In this study, excluding the possible error of sampling by bronchoscopy, we systematically evaluated the ability of LungMe^®^ to diagnose lung cancer in FFPE tissue samples. The results manifest for the first time that, the assessment of *SHOX2* and *RASSF1A* methylation levels in FFPE tissue sample can improve the accuracy of lung cancer diagnosis compared with conventional pathology alone. Our observation indicated that *SHOX2* and *RASSF1A* may play different roles in the process of cancer development. The combined use of *SHOX2* and *RASSF1A* methylation detection greatly improved sensitivity, which provides us with a more accurate method of differential diagnosis, and it is likely to be rapidly applied in clinical examinations.

## Materials and Methods

### Patients and Specimens

This study was approved by the Ethics Committee of Tongji Hospital of Tongji University. The registration number of this clinical study is KYSB-2018 (048). All of samples were obtained from consenting individuals according to protocols approved by the ethics committee. The patients/participants provided their written informed consent to participate in this study.

Left-over FFPE resection specimens from 251 patients were examined in our study. Among them, 137 cases were diagnosed as lung cancer, including 70 adenocarcinoma (I:40, II: 10, III:10, IV:10), 51 squamous cell carcinoma (I:17, II:13, III:12, IV:9) and 16 small cell lung cancer (III:8, IV:8). The other 114 cases were benign diseases as control, including inflammatory infection, tuberculous granuloma, and other granulomatous tissues. All 251 FFPE specimens were randomly divided into the training set (n = 135) and validation set (n = 116), which were used in order to transfer the clinical decision point to the test kit (**Table 1**). Thirty pairs of cancer tissues and paracancerous tissue specimens were used for clinical validation. The cancer samples were histologically viable tumor. Their paracancerous tissue samples were the adjacent normal tissues 1–2 cm away from the malignant lesion without histologically viable tumor cells. In additional, 17 puncture biopsy specimens from lung cancer patients, in which small mammary focus were not detected by conventional pathologic evaluation, were analyzed for *SHOX2* and *RASSF1A* DNA methylation levels.

**Table 1:** Clinical information of the training set and validation set.

Characteristics	Tissue Sample (FFPE)									
	Training set					Validation set				
	Lung cancer			Control		Lung cancer			Control	
	Adenoca(n = 35)	Squamous(n = 30)	SCLC(n = 10)	Benign(n = 60)	Total(n = 135)	Adenoca(n = 35)	Squamous(n = 21)	SCLC(n = 6)	Benign(n = 54)	Total(n = 116)
Age (years)										
Mean ± SEM	55.8 ± 2.1	62.0 ± 1.5	64.1 ± 1.4	56.6 ± 1.9	61.8 ± 0.9	53.5 ± 2.3	60.2 ± 1.6	61.2 ± 1.4	59.7 ± 1.7	60.7 ± 0.9
Range	34-72	44–76	31–79	44–76	31–79	35–80	43–80	39–80	34–80	34–80
Gender										
Female (%)	15(42.9)	8(28.7)	4(40)	25(41.7)	52(38.5)	18(51.4)	9(42.9)	2(33.3)	21(38.9)	50(43.1)
Male (%)	20(57.1)	22(73.3)	6(60)	35(58.3)	83(61.5)	17(48.6)	12(57.1)	4(66.7)	33(61.1)	66(56.9)
Stage										
Stage I (%)	20 (57.1)	10 (33.3)	–			20 (57.1)	7(33.3)	–		
Stage II (%)	5 (14.3)	8 (26.7)	–	–	75(55.6)	5 (14.3)	5(23.8)	–	–	62(53.4)
Stage III (%)	5 (14.3)	7 (23.3)	5 (50.0)			5 (14.3)	5(23.8)	3 (50.0)		
Stage IV (%)	5 (14.3)	5 (16.7)	5 (50.0)			5 (14.3)	4(19.0)	3 (50.0)		
Benign	–			60	60(44.4)	–			54	54(46.6)

All FFPE samples were not being stored for more than 2 years. The diagnosis was confirmed on the basis of histological results, and the tumor stages were determined according to the American Joint Committee on Cancer (AJCC) staging system as revised in 2010 (7th edition).

### Analytical Performance: Reproducibility and Accuracy

A series of model samples with deﬁned methylated *SHOX2* levels (0.4, 0.8, 1.6, 25, and 100%) were prepared by mixing bisulﬁte-converted methylated SHOX2 DNA plasmid with bisulﬁte-converted DNA from sperm cells which were unmethylated at the *SHOX2* locus. A series of model samples with deﬁned methylated *RASSF1A* levels (0.4, 0.8, 1.6, 25, 100%) were prepared by mixing bisulﬁte-converted methylated RASSF1A DNA plasmid with bisulﬁte-converted DNA from Hela cells which were not shown methylated at the *RASSF1A* locus. In addition, three model samples for each *SHOX2* and *RASSF1A* were prepared by spiking three different levels of total DNA concentration indicated by the reference gene Ct*_β_*_-ACTB_ (Ct*_β_*_-ACTB_ = 18:10,000 copies, Ct*_β_*_-ACTB_ = 20:2,500copies, Ct*_β_*_-ACTB_ = 23:625copies).

The DNA concentration of sperm cells, Hela cells and bisulﬁte-converted methylated *SHOX2* and *RASSF1A* DNA plasmids were determined by using the highly sensitive Qubit assays (Qubit 3/4 Fluorometer, High sensitive fluorescent dye).

### Determination of *SHOX2* and *RASSF1A* DNA Methylation Levels in FFPE Specimens Using the LungMe^®^ Assay

The DNA methylation levels of *SHOX2* and *RASSF1A* in FFPE specimens were determined using the NMPA (China National Medical Products Administration) marked *in vitro* diagnostic (IVD) test LungMe^®^ assay (20173403354, Tellgen Co., China).

The FFPE DNA extraction kit (CWY009S FFPE DNA Kit, CW Biotech Co., Ltd., China) was used for the lysis of paraffin-embedded tissue material. The concentration of extracted DNA was accurately measured using the highly sensitive Qubit assays (Qubit 3/4 Fluorometer, High sensitive fluorescent dye). Fifty ng DNA/sample was treated with sodium bisulfite using the Tellgen DNA purification kit (PF03X056, Tellgen Co., China). After purification, the bisulfite-converted DNA was amplified by methylation specific real-time PCR (MA-PCR) amplification using LungMe^®^ real-time PCR Kit (as described before) (11, 12). The MS-PCR amplifies methylated *SHOX2*(VIC), *RASSF1A*(FAM), and *β-ACTB*(CY5) DNA which served as a reference for the quantification of total input DNA. The positive quality controls are plasmids containing the methylated DNA of *SHOX2* and *RASSF1A* that have no bioactivity. PCR amplification was performed in an ABI 7500 Real-Time PCR instrument (Applied Biosystems, CA, UAS), and SDS Software (Applied Biosystems) was selected to conduct the results of the analysis. The relative amount of methylated *SHOX2* and *RASSF1A* was calculated to the delta cycle threshold ΔCt (ΔCt*_SHOX2_* = Ct*_SHOX2_* − Ct*_β_*_-ACTB_; ΔCt*_RASSF1A_* = Ct*_RASSF1A_* − Ct*_β_*_-ACTB_) method.

### Establishment of Methylation Cut-Off for Patient Stratification

The training set comprising a total of 135 FFPE specimens (75 cases and 60 controls) was used in order to transfer the clinical decision point to the test kit. The receiver operating characteristic (ROC) curves are performed to determine the cut-off values of ΔCt*_SHOX2_* and ΔCt*_RASSF1A_* for the diagnosis of lung cancer.

## Results

### Analytical Performance: Reproducibility and Accuracy

**Figure 1:**
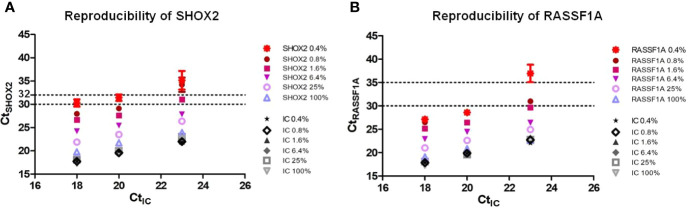
Reproducibility of Lung-Me^®^ Assay **(A)**. Reproducibility of *SHOX2* was evaluated by analyzing six different relative *SHOX2* concentrations (0.4, 0.8, 1.6, 6.4, 25, and 100%) with three different levels of total DNA concentration indicated by CtIC (18:10,000 copies, 20:2,500 copies, 23:625 copies) **(B)**. Reproducibility of *RASSF1A* was evaluated by analyzing five different relative *RASSF1A* concentrations (0.1, 0.4, 0.8, 6.4, 25, and 100%) with three different levels of total DNA concentration indicated by CtIC (18:10,000 copies, 20:2,500 copies, 23:625 copies). Five PCR replicates pre sample were performed. To assess reproducibility, mean Ct, SD, %CV was calculated (assign “NoCt” = 40).

The detection of gene methylation needs to consider both relative concentration and absolute copy number of this gene. Therefore, the reproducibility of *SHOX2* and *RASSF1A* was separately evaluated by analyzing six different relative *SHOX2* or *RASSF1A* concentrations (0.4, 0.8, 1.6, 6.4, 25, 100%) under the background of three different levels of unmethylated total DNA (10,000 copies, 2,500 copies, 625 copies) prepared by sperm cells or Hela cells, respectively. Five PCR replicates pre r sample were performed. To assess reproducibility, mean Ct, SD, %CV were calculated (assign “NoCt” = 40). The analytical performance of the assay is shown in **Figure 1** (data were shown in **Supplementary Table 1**). As shown in **Figure 1**, with the exception of five pre-cycle, when the Ct-value of the MS-PCR is within 32 for SHOX2-VIC channel, the average coefficient variation (CV) is between 0.5 and 5.7%. When the Ct-value of the MS-PCR is within 35 for the RASSF1A-FAM channel, the average coefficient variation (CV) is between 0.2 and 2.3%. Therefore, the assay allowed for the reliable detection of *SHOX2* DNA methylation with cycle threshold values under 32, while it can reliably detect *RASSF1A* DNA methylation within 35.

Under the background of 10,000 copies of unmethylated total DNA (Ct*_β_*_-ACTB_ = 18), the detection limitation of the assay for *SHOX2* and *RASSF1A* DNA is 0.4%, 40 copies. Under the background of 2,500 copies unmethylated total DNA (Ct*_β_*_-ACTB_ = 20), the detection limit of the assay for *SHOX2* and *RASSF1A* DNA is 0.4%, 10 copies. Under the background of 625 copies unmethylated total DNA (Ct*_β_*_-ACTB_ = 23), the detection limitation of the assay for *SHOX2* is 1.6%, 10 copies, while for *RASSF1A* is 0.8%, five copies.

### Clinical Performance

To establish a clinical cut-off for patient stratification, firstly, MS-PCR was used to quantify the DNA methylation in the training set comprising a total of 135 FFPE samples (75 cases and 60 benign controls). The relative amount of methylated *SHOX2* and *RASSF1A* were calculated for each sample, respectively, according to the delta cycle threshold (ΔCt) method. Valid results according to the instructions for use (18≤Ct*_β_*_-ACTB_<32 for a valid positive result whereas 18≤Ct*_β_*_-ACTB_ ≤23 for a valid negative result). According to the reproducibility of the assay, the Ct*_SHOX2_*<32 and Ct*_RASSF1A_*<35 criterions were also set for calculating ΔCt. A receiver operating characteristic plot (ROC) analysis in the training set was performed, where the area under the ROC curve (AUC) was calculated and cut-off value was determined accordingly. The sensitivity and specificity were further calculated based on this cut-off. Moreover, to robustly estimate the diagnostic accuracy, an independent evaluation using the validation set (62 cases and 54 benign controls) was performed, where another sensitivity and specificity were calculated based on the given cut-off. With a methylation cut-off of ΔCt*_SHOX2 =_*7.5, the specificity and sensitivity of *SHOX2* were 91.7 and 76.0% in the training set and were 92.6 and 80.6% in the validation set. With a methylation cut-off of ΔCt*_RASSF1A_*=12, the specificity and sensitivity of *RASSF1A* were 93.3 and 61.3% in the training set and were 98.1 and 61.3% in the validation set. Combining *SHOX2* and *RASSF1A*, the specificity and sensitivity of LungMe^®^ were 90.0 and 89.3% in the training set and were 90.7 and 90.3% in the validation set, indicating that SHOX2 and RASSF1A gene methylation as lung cancer biomarker panel has excellent accuracy for lung cancer diagnosis (data were shown in **Supplementary Table 2**).

For all samples (comprising the training and validation data set), the clinical performance with regard to pathologically determined histological classification and tumor stage was analyzed and listed in **Table 2**. The positive detection rates of LungMe^®^ in SCLC, squamous carcinoma, adenocarcinoma and total were 100.0, 96.1, 82.9, and 89.8%. In the control group, 11 of 114 patients with benign lung diseases were detected as *SHOX2* or *RASSF1A* positive, resulting in a specificity of 90.4%. Overall, the methylation analysis of *SHOX2* in FFPE showed a higher detection sensitivity (78.1%) and relative lower specificity (92.1%) compared with the sensitivity (61.3%) and specificity (95.6%) of *RASSF1A*. Furthermore, *SHOX2* alone allows for the detection of SCC with a high sensitivity of 94.1%. Combine with *RASSF1A*, the detection rates in adenocarcinoma and SCLC were greatly improved from 64.3 to 82.9% and from 87.5 to 100%. The lower sensitivity of *SHOX2* was only observed with stage I adenocarcinoma, which was greatly improved by adding *RASSF1A* (increased from 42.5 to 70%). However, the positive detection rates of *SHOX2* alone for stage II adenocarcinoma and Stage I SCLC were 80.0 and 88.2%, respectively.

**Table 2:** Detection sensitivity of *SHOX2* and *RASSF1A* methylation in different histological subtype groups and tumor stage groups.

Tumor Classification	SHOX2	RASSF1A	Both methylated	Either mehtylated
	n(%)	n(%)	n(%)	n(%)
Lung cancer				
Histology and Stage				
Adenoca (n = 70)	45(64.3)	43(61.4)	30(42.9)	58(82.9)
Stage I (n = 40)	17(42.5)	24(60.0)	13(32.5)	28(70.0)
Stage II (n = 10)	8(80.0)	6(60.0)	4(40.0)	10(100.0)
Stage III (n = 10)	10(100.0)	7(70.0)	7(70.0)	10(100.0)
Stage IV (n = 10)	10(100.0)	6(60.0)	6(60.0)	10(100.0)
Squamous (n = 51)	48 (94.1)	28(54.9)	27(52.9)	49(96.1)
Stage I (n = 17)	15(88.2)	13(76.5)	12(70.6)	16(94.1)
Stage II (n = 13)	13(100.0)	7(53.8)	7(53.8)	13(100.0)
Stage III (n = 12)	11(91.7)	6(50.0)	6(50.0)	11(91.7)
Stage IV (n = 9)	9(100.0)	2(22.2)	1(22.2)	9(100.0)
SCLC (n = 16)	14(87.5)	13(81.3)	11(68.8)	16(100.0)
Stage III (n = 8)	7(87.5)	6(75.0)	5(62.5)	8(100.0)
Stage IV (n = 8)	7(87.5)	7(87.5)	6(75.0)	8(100.0)
Total (n = 137)	107 (76.6)	84(61.3)	68(51.1)	123(89.8)
Control				
Benign (n = 114)	9(7.9)	5(4.4)	3(2.6)	11(9.6)

**Figure 2:**
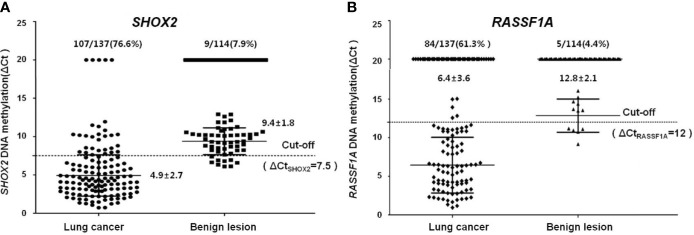
Quantitative analyzing of *SHOX2* **(A)** and RASSF1A **(B)** DNA methylation in different histology groups **(A)**. *SHOX2* DNA methylation values measured in lung cancer (dots) and benign lesion (squares) **(B)**. *RASSF1A* DNA methylation values measured in lung cancer (diamonds) and benign lesion (triangles).

**Figure 3:**
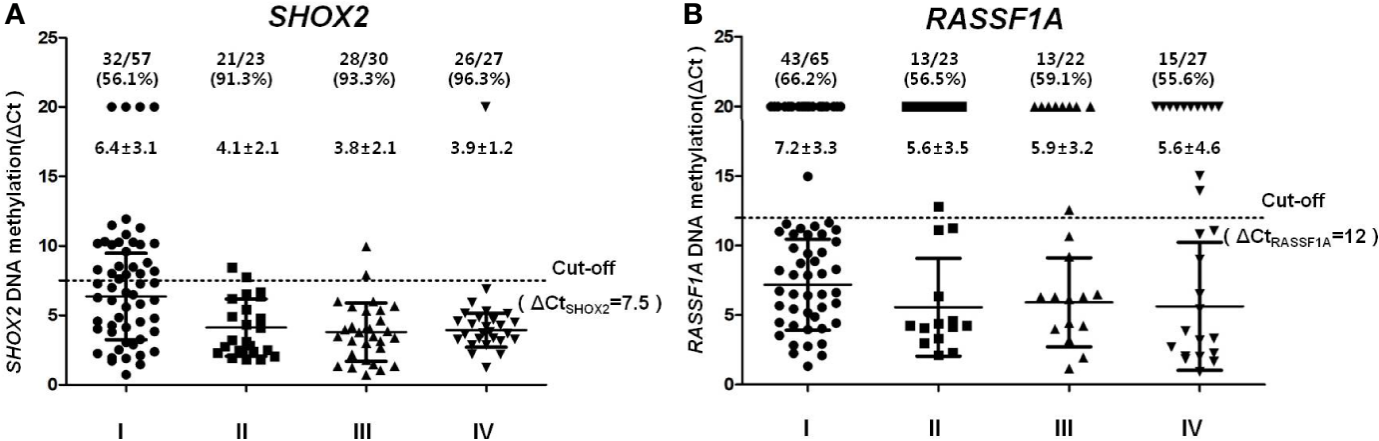
Quantitative analysis of *SHOX2* **(A)** and *RASSF1A* **(B)** DNA methylation in different tumor stage groups **(A)**. *SHOX2* and **(B)** *RASSF1A* DNA methylation values measured in different tumor stage, I (dots), II (squares), III (up-triangles), IV (down-triangles).

The association between clinicopathological features (lung cancer and benign lesion) and quantitative *SHOX2* and *RASSF1A* methylation status (ΔCt*_SHOX2_* and ΔCt*_RASSF1A_*) was analyzed in more details and plotted in **Figure 2**. As shown in **Figure 2**, the significantly higher *SHOX2* (A, 4.9 ± 2.7) and *RASSF1A* (B: 6.5 ± 3.6) DNA methylation (lower ΔCts) can be found in FFPE samples from lung cancer cases in comparison to the benign lesion controls (A, 9.4 ± 0.8; B, 12.9 ± 2.1). These results indicated that the level of *SHOX2* methylation increased by ~20-fold and the level of *RASSF1A* methylation increased by ~80-fold in positive cancer specimens. Furthermore, from stages I to IV, the positive detection rate of *SHOX2* hypermethylation notably increased from 76.6% in stage I (ΔCt = 6.4 ± 3.1) to 91.3% in stage II [Δ(o=4.1 ± 2.1)], 93.3% in stage III (ΔCt = 3.8 ± 2.1) and 96.3% in stage IV (ΔCt = 3.9 ± 31.2). However, *RASSF1A* has no significant differences among stages of cancer, stage I (ΔCt = 7.2 ± 3.3) is 66.2%, stage II (ΔCt = 5.6 ± 3.5) is 56.5%, stage III (ΔCt = 5.9 ± 3.2) is 59.1% and stage IV (ΔCt = 5.6 ± 4.6) is 55.6%. This quantitative analysis in **Figure 3** shows that the degree of *SHOX2* methylation is correlated with the stages of lung cancer, whereas the correlation is not observed in the case of *RASSF1A* methylation.

### Clinical Validation: Paired Lung Cancer and Cancer-Adjacent Specimen

**Figure 4:**
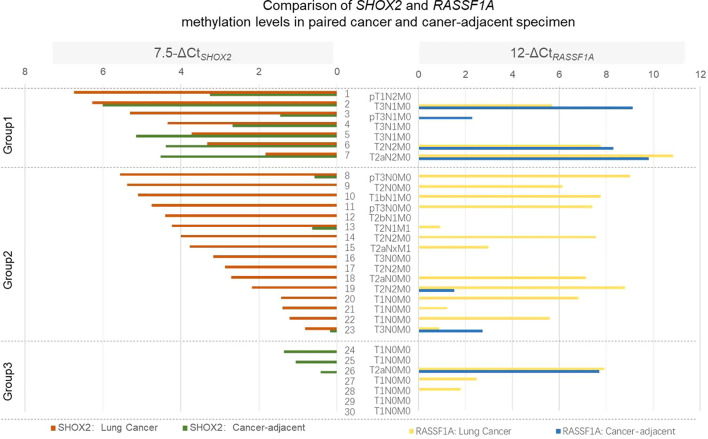
Comparison of *SHOX2* and *RASSF1A* methylation levels in paired lung cancer and cancer-adjacent specimen. *SHOX2* and *RASSF1A* methylation levels were detected in paired lung cancer and cancer-adjacent specimen. In order to display the change of methylation level more intuitively, the methylation levels of *SHOX2* and *RASSF1A* were displayed as 7.5-ΔCt*_SHOX2_* and 12-ΔCt*_RASSF1A_*, respectively. Based on *SHOX2* methylation level in cancer specimen, the results were organized in three groups.

Initial pathology analysis confirmed 30 pairs of lung cancer and adjacent tissues. Twenty-three cancer tissues and 13 of cancer-adjacent specimens were classified as *SHOX2* methylation positive with ΔCt*_SHOX2_* ≤7.5. However, 19 of cancer tissues and seven of cancer-adjacent specimens were classified as *RASSF1A* methylation positive with ΔCt*_RASSF1A_* ≤12. The comparison of *SHOX2* and *RASSF1A* methylation levels in matched lung cancer and cancer-adjacent specimen was shown in **Figure 4**. In order to display the change of methylation level more intuitively, the methylation levels of *SHOX2* and *RASSF1A* were displayed as 7.5-ΔCt*_SHOX2_* and 12-ΔCt*_RASSF1A_*, respectively. Based on *SHOX2* methylation level in cancer specimens, the results were organized in three groups. In group 1, high levels of *SHOX2* methylation were observed in both cancer and cancer-adjacent specimens; besides, what were really noticeable were all seven cases in this group with at least one lymph node metastasis. In group 2, the significant reduction in *SHOX2* methylation between cancer and cancer-adjacent specimen was observed in 16 cases. In group 3, five of seven early stage I cancer-adjacent but not cancer specimen exhibited the hypermethylation of *SHOX2* or *RASSF1A*. A possible explanation for this could be the low cancer cells’ proportion in the left-over FFPE specimen of early small lesions. The change of *RASSF1A* methylation level shows a similar trend but not exactly the same in comparison with *SHOX2*. The results of this study revealed that, on the one hand, the hypermethylation of *SHOX2* and *RASSF1A* has a high degree of tumor specificity. On the other hand, cells adjacent to cancer foci can contain DNA methylation changes, which may be indistinguishable by histopathology, but detectable by methylation specific PCR testing, which was so-called “Epigenetic Field Effect”.

### Clinical Validation: Pairs of Negative Puncture Biopsy and Positive Surgical Operative Specimen Detected by Conventional Pathologic Evaluation

**Figure 5:**
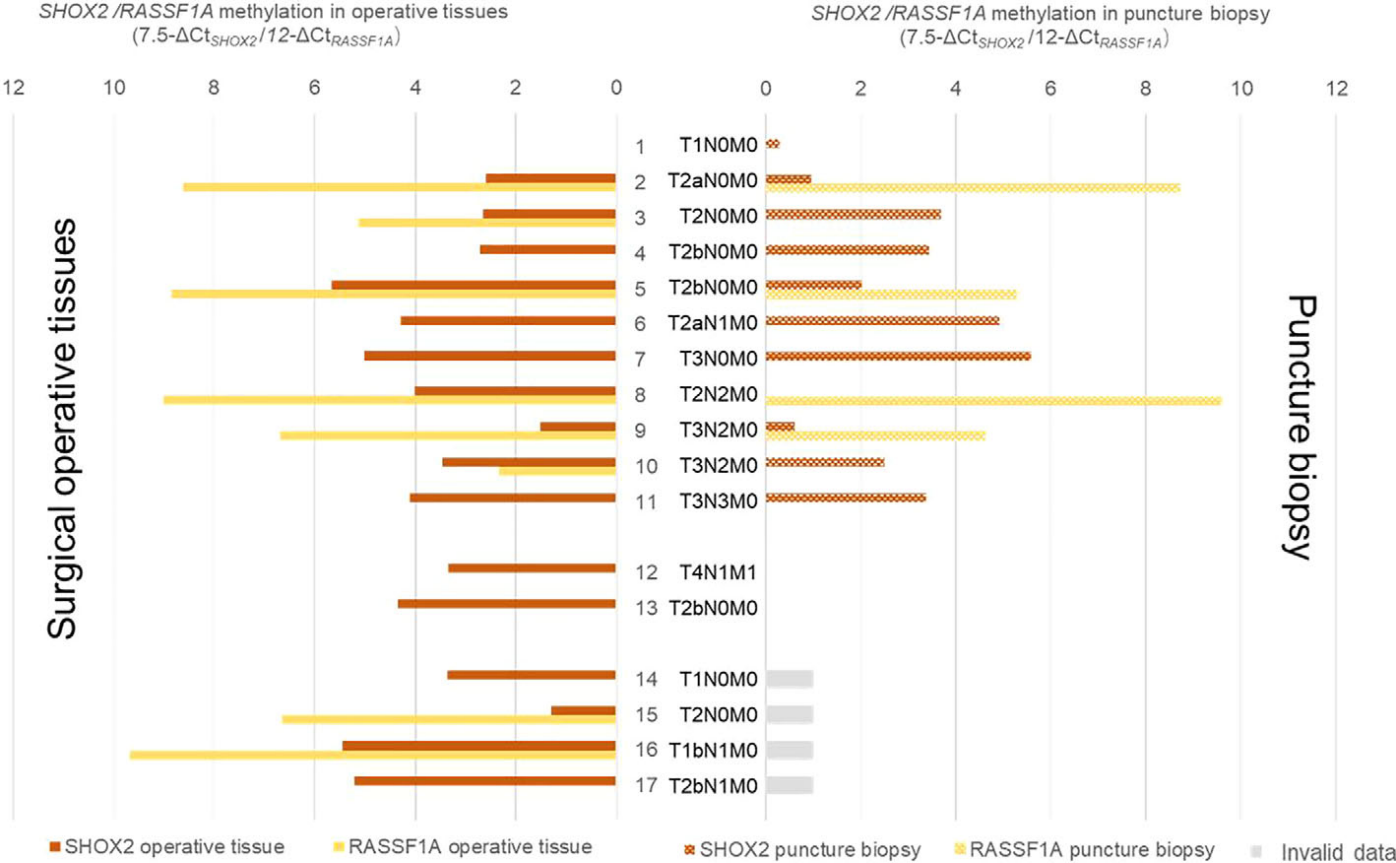
Comparison of *SHOX2* and *RASSF1A* methylation levels in paired negative puncture biopsy and positive surgical operative specimen detected by conventional pathologic evaluation. *SHOX2* and *RASSF1A* methylation levels were detected in 17 paired lung cancer negative puncture biopsy and positive surgical operative specimen. In order to display the change of methylation level more intuitively, the methylation levels of *SHOX2* and *RASSF1A* were displayed as 7.5-ΔCt*_SHOX2_* and 12-ΔCt*_RASSF1A_*, respectively.

In additional 17 puncture biopsy specimens from lung cancer patients, which focus on small mammary were not detected by conventional pathologic evaluation, together with their pathologic analysis confirmed surgical operative specimen were analyzed for *SHOX2* and *RASSF1A* DNA methylation levels. As shown in **Figure 5**, the hypermethylation of *SHOX2* or *RASSF1A* was detected not only in 16 of these surgical operative specimens, but also in 11 of those histopathological negative puncture biopsies. In the remaining six puncture biopsies, ΔCts of *SHOX2* and *RASSF1A* was found to be below the cut-off. Two of them were classified as methylation negative, whereas four of them were classified as detection invalid due to insufficient DNA concentration (Ct*_β_*_-ACTB_>23). Comparing the LungMe^®^ results with the pathologic results, the methylation biomarkers reached a much higher sensitivity and could significantly improve the diagnostic efficacy.

## Discussion

At present, the conventional pathological diagnosis to a large extent belongs to “empirical science”, which is highly subjective. The pathological diagnosis, including cyto- and histopathological examinations, could be severely affected by the quality of specimen and the diagnostic level of the individual pathologists (3, 4). The sensitivity and repeatability of the detection need to be improved and enhanced. Emerging molecular diagnosis methods are more sensitive and objective, which can overcome the intrinsic failings of morphological diagnosis. DNA methylation alterations, among the most promising biomarkers in early diagnosis of cancer, have been transferred from scientific research to clinical application with a growing number (5, 15). Many of researches have reported several targeted DNA methylation biomarkers and confirmed the role in the diagnosis of lung cancer (6, 10, 16, 17). The NMPA (China National Medical Products Administration) marked *in vitro* diagnostic (IVD) test ‘LungMe^®^’, has been developed and confirmed having a good diagnostic effect, with a sensitivity of 71.5–81.0%, and a specificity of 90–97.4% (11, 12). In this study, excluding the possible error of sampling by bronchoscopy, we systematically evaluated the ability of LungMe^®^ to diagnose lung cancer in FFPE tissue samples.

The detection limit of gene methylation is determined by the relative concentration and the absolute copy number of this gene in the assay testing system. Therefore, firstly through the quantification of DNA, the detection amount of 50 ng DNA in each test is guaranteed to ensure the diagnostic sensitivity of each test. Secondly, the calculation method of ΔCt is used to increase the repeatability of detection and the comparability between samples. Thirdly, the detection and analysis should be within the stable detection range of the assay. Our data shows that the LungMe^®^ assay allowed for the reliable detection of methylated SHOX2 DNA with cycle threshold values under 32 (CV between 0.5 and 5.7%), while the reliable detection of RASSF1A DNA is within 35 (CV between 0.2 and 2.3%). As 18≤Ct*_β_*_-ACTB_ ≤23, the detection limit of *SHOX2* is 10–40 copies (0.4–1.6%), while the detection limit of *RASSF1A* is 5–40 copies (0.4–0.8%). Fourthly, the receiver operating characteristic (ROC) curves are performed to determine the cut-off values of ΔCt*_SHOX2_* and ΔCt*_RASSF1A_* for lung cancer diagnosis. With the methylation cut-off of ΔCt*_SHOX2 =_*7.5 and ΔCt*_RASSF1A_* =12, *SHOX2* in FFPE showed a higher detection sensitivity (76.6%) and relatively lower specificity (92.1%) when compared with *RASSF1A* sensitivity (61.3%) and specificity (95.6%). The supplement of *RASSF1A* to *SHOX2* greatly improves the sensitivity of diagnosis from 76.6 to 89.8%, but it still ensures a 90.4% specificity of the combination. LungMe^®^ is the worldwide first commercialized methylation detection kit comprising *SHOX2* and *RASSF1A* panel for lung cancer detection. According to this optimized detection process, the standardized DNA input ensures that the Ct*_β_*_-ACTB_ value (around 20) fluctuates within a small range, thereby ensuring the sensitivity of each test. More importantly, adding the objective criteria, Ct*_β_*_-ACTB_ >23 for an invalid negative result, let us be more confident about negative results.

Previous studies based on analysis of bronchial lavage samples have already showed that the positive detection rates of the LungMe^®^ panel in LCLC, SCLC, SCC, and adenocarcinoma were 100, 87.5–90.5, 82.4–88.8 and 54.9–69.6%, respectively (12, 15). Adenocarcinoma exhibited the lowest positive detection rate, which was consistent with the research results with other detection kits (6, 18). Small lesions and mostly peripheral types are the major causes for the miss sampling of adenocarcinoma especially at early stage by bronchoscopy. Therefore, the design of this study focused on the early stage of adenocarcinoma. The left-over FFPE specimens of 251 patients (137 cases, 114 controls) were examined in our study. Among them, 137 were diagnosed as lung cancer, including 70 (51%) adenocarcinoma, 51 (37%) SCC, and 16 (12%) SCLC, namely, 57 (41%) cases of stage I, 23 (17%) cases of stage II, 30 (22%) cases of stage III, and 27 (20%) cases of stage IV. In our study, based on FFPE samples, the positive detection rates of the LungMe^®^ panel in SCLC, SCC, and adenocarcinoma were 100, 96.1, and 82.9%. Furthermore, *SHOX2* alone allows for the detection of SCC with high sensitivity of 94.1%. Combined with *RASSF1A*, the detection rates in adenocarcinoma and SCLC were greatly improved from 64.3 to 82.9% and from 87.5 to 100%. Notably, the positive detection rate of *RASSF1A* was 61.4% in adenocarcinoma and 54.9% in SCC, which was significantly improved in comparison to other study in histology specimens, 39% in adenocarcinoma and 13% in SCC, respectively (19).

The lower sensitivity of *SHOX2* was only observed with stage I adenocarcinoma, which was greatly improved by adding *RASSF1A* (increased from 42.5 to 70%). However, the positive detection rates of *SHOX2* alone for stage II adenocarcinoma, and stage I SCC were 80 and 88.2%, respectively. For small lesion at early stage, microdissection can also improve the proportion of tumor cells and the sensitivity of diagnosis. Furthermore, our data shows that from stage I to stages II, III, IV, both the positive detection rate and the methylation level of *SHOX2* were rapidly increased from 56.1% (ΔCt = 6.4 ± 3.1) to 91.3% (ΔCt = 4.1 ± 2.1), 93.3% (ΔCt = 3.8 ± 2.1) and 96.3% (ΔCt = 3.9 ± 31.2), However, the detection sensitivity of *RASSF1A* for stages I to IV were 66.2% (ΔCt = 7.2 ± 3.3), 60.9% (ΔCt = 5.6 ± 3.5), 63.6% (ΔCt = 5.9 ± 3.2) and 55.6% (ΔCt = 5.6 ± 4.6). This quantitative analysis shows that the degree of *SHOX2* methylation is correlated with the stage of lung cancer, whereas the correlation is not observed in the case of *RASSF1A* methylation. Since *SHOX2* and *RASSF1A* are simultaneously detected in the same reaction tube, the influence of sample error on experimental results can be completely excluded. Interestingly, Yu et al. demonstrated that hypermethylated *HISTIH4F* could serve as a pan-cancer biomarker and also observed the *HIST1H4F* methylation without significant differences among stages of cancer (20). The high frequency of *RASSF1A* promoter methylation has a high correlation with cancer pathogenesis and a more aggressive clinical phenotype (21, 22). Our observation indicated that *SHOX2* and *RASSF1A* might play different roles in initiation, proliferation, invasion, and metastasis of cancer development.

Next, the methylation levels of *SHOX2* and *RASSF1A* in 30 pairs of cancer and caner-adjacent tissues were analyzed. Our data showed that 23 of cancer tissues and 13 of cancer-adjacent specimens were classified as *SHOX2* methylation positive, whereas 19 of cancer tissues and seven of cancer-adjacent specimens were classified as *RASSF1A* methylation positive. In most of the cases (group 2), the significant reduction in the methylation level of *SHOX2* and/or RASSF1A between cancer and cancer-adjacent specimen was observed. This indicated that the hypermethylation of *SHOX2* and *RASSF1A* was highly tumor-specific. More importantly, in group 1, seven cases with lymph node metastasis exhibited high *SHOX2* methylation level in both cancer and cancer-adjacent specimen, whereby cells adjacent to cancer foci can contain DNA methylation changes, which may be indistinguishable by histopathology, but detectable by methylation specific PCR testing. Field defects often appear to be histologically normal under the microscope. Previous research indicates that cells within a field defect characteristically have an increased frequency of epigenetic alterations and these may be fundamentally important as underlying factors in progression to cancer (23).

Furthermore, we investigated that whether the diagnosis of LungMe^®^ can be applied to a miss-sampling that didn’t hit the bull’s eye. As a final step in the diagnosis of lung cancer, it has been confirmed that histopathological specimens obtained from transbronchial (TBLB), endobronchial biopsy (EBLB), and transthoracic needle aspiration (TTNA) were used for diagnosing and staging of lung cancer with a sensitivity of 71–98% (2, 13, 14). However, the diagnostic performance depends on individual experience of investigators including bronchoscopists and pathologists. Besides, due to possible sampling error, the risk of missing a malignant lesion can never be excluded by any test. The advantages of DNA methylation detection are more objective, more sensitive, and more wide-ranging. Based on our results in this study, the assessment of *SHOX2* and *RASSF1A* DNA methylation identified 11 of 17 malignant puncture biopsy specimens with no signs of malignancy by conventional pathology were referred to surgical biopsies, which were confirmed with lung cancer, making this assay an applicable and relative fast method to detect malignant in puncture biopsy specimens and improved sensitivity of lung cancer detection with small biopsy specimens.

We expected to improve the validation rate of LungMe^®^, with implementation of the assay and increase laboratory experience. Whether the diagnosis of LungMe^®^ can be applied to the judgment of surgical margins and the diagnosis of lymph nodes (7), could be the next research direction in the future.

The presented results show for the first time that the assessment of *SHOX2* and *RASSF1A* methylation levels in FFPE tissue samples can improve the accuracy of lung cancer diagnosis compared with conventional pathological alone. Our observation revealed that *SHOX2* and *RASSF1A* may play different roles in initiation, proliferation, invasion, and metastasis of cancer development. The *SHOX2* and *RASSF1A* methylation detection greatly improves the sensitivity of lung cancer detection. Early recognition of these entities produces a more accurate differential diagnosis and may enable more expeditious clinical workup.

## Data Availability Statement

The original contributions presented in the study are included in the article/**Supplementary Materials**; further inquiries can be directed to the corresponding authors.

## Ethics Statement

This study was approved by the ethics committee of Tongji Hospital of Tongji University. The registration number of this clinical study is KYSB-2018(048). The patients/participants provided their written informed consent to participate in this study.

## Author Contributions

BS, HH, and XY, as the co-corresponding authors, contributed to the conception of the study, and significantly to the analysis and manuscript preparation, and wrote the manuscript and helped perform the analysis with constructive discussions. JS, XC, and LZ and the first author performed the experiments and analyzed the data. All authors contributed to the article and approved the submitted version.

## Funding

This study was supported by the National Natural Science Foundation of China (81600043, 81800063), Shanghai Natural Science Foundation (16ZR1432100, 19ZR1448500), Shanghai Health Bureau Key Special Fund for Medicine (20134034), and Shanghai Science and Technology Commission Medical Guidance project fund (19411964700).

## Conflict of Interest

BS was employed by the company Tellgen Corporation, Shanghai, China.

The remaining authors declare that the research was conducted in the absence of any commercial or financial relationships that could be construed as a potential conflict of interest.
